# Systemic Immune Alterations in Paediatric Classical Hodgkin Lymphoma With CCL17 and MCP‐4 as Diagnostic and Predictive Biomarkers

**DOI:** 10.1002/jha2.70228

**Published:** 2026-01-28

**Authors:** Gustav Hedberg, Qi Chen, Tadepally Lakshmikanth, Nikolas Herold, Per Kogner, Petter Brodin, Linda Ljungblad

**Affiliations:** ^1^ Childhood Cancer Research Unit Department of Women's and Children's Health Karolinska Institutet Stockholm Sweden; ^2^ Clinical Pediatrics Unit Department of Women's and Children's Health Karolinska Institutet Stockholm Sweden; ^3^ Department of Immunology and Inflammation Imperial College London London UK; ^4^ Medical Research Council Laboratory of Medical Sciences Imperial College Hammersmith Campus London UK; ^5^ Section Pediatric Oncology Astrid Lindgren Children's Hospital Karolinska University Hospital Stockholm Sweden; ^6^ Pediatric Rheumatology Unit Astrid Lindgren Children's Hospital Karolinska University Hospital Stockholm Sweden; ^7^ Theme Cancer Karolinska University Hospital Stockholm Sweden

**Keywords:** chemotherapy, neutropenic fever, paediatric oncology, solid tumours, systems‐level immunomonitoring

## Abstract

**Background:**

Paediatric classical Hodgkin lymphoma (cHL) is the most common malignancy in adolescents, and despite excellent survival, a subset of patients experiences treatment failure or severe long‐term toxicity, underscoring the need for improved risk stratification. Early response assessment is particularly important, as it guides decisions on radiotherapy, where overtreatment can lead to substantial late effects.

**Methods:**

In the context of a large‐scale systems‐level immunomonitoring initiative, we specifically examined paediatric cHL and profiled their systemic immunology alongside children with intra‐ and extracranial solid tumours and other lymphomas. Through longitudinal sampling before and after treatment, we aimed to identify diagnostic and prognostic biomarkers relating immune profiles to early treatment response and risk of developing neutropenic fever.

**Results:**

Plasma CCL17 and MCP‐4 were markedly elevated in cHL compared with other paediatric lymphomas and other solid tumours, with distinct immune cell compositions, particularly between cHL and extracranial tumours. CCL17 and MCP‐4 negatively correlated with age in extracranial and intracranial tumours but not in cHL, indicating disease‐specific regulation. Chemotherapy induced consistent protein changes in cHL and eliminated CCL17 and MCP‐4 differences between cHL and other lymphomas. Lower baseline MCP‐4 and greater CCL17 reduction after chemotherapy were associated with favourable early response, while lower granzyme levels identified patients at higher neutropenic fever risk.

**Conclusion:**

Together, these exploratory findings highlight clinically relevant biomarkers in a paediatric oncology context, with the potential to enhance diagnostic precision, guide response‐adapted therapy and effectively allocate supportive care, thereby improving outcomes for children with cHL.

**Trial Registration:**

The authors have confirmed clinical trial registration is not needed for this submission

**Abbreviations**
ANC: absolute neutrophil count
CCL17: C‐C motif chemokine 17
cHL: classical HL
DP T‐cells: double positive T‐cells
HL: Hodgkin lymphoma
HRS: Hodgkin and Reed‐Sternberg
MCP‐4: monocyte chemoattractant protein‐4
mDC: myeloid dendritic cell
NLPHL: nodular lymphocyte‐predominant HL
PCA: principal component analysis
PET/CT: positron emission tomography‐computed tomography
Treg: T‐regulatory cell
WBC: white blood cell count

## 1 Introduction

Paediatric classical Hodgkin lymphoma (cHL) is the most common malignancy in adolescents and despite high overall survival, treatment failure occurs in 15%–20% of advanced‐staged patients and standard treatment comes with severe toxicity [1, 2]. There are two main subtypes of Hodgkin lymphoma (HL), classical HL (cHL) and nodular lymphocyte‐predominant HL (NLPHL), with the vast majority being cHL, which differ from NLPHL in histology, immunophenotype and treatment strategy [2, 3, 4]. Classical HL is a B‐cell lymphoma characterised by a minority of malignant Hodgkin and Reed‐Sternberg (HRS) cells surrounded by non‐cancerous stromal and immune cells [5, 6]. While local tumour immunobiology is well described, systemic immune responses in children with cHL and their modulation by chemotherapy remain poorly understood.

Due to the rarity of malignant HRS cells, diagnosing cHL relies on excisional lymph node biopsy [2], a sometimes complicated procedure requiring specialised expertise [7]. Blood‐based biomarkers are easy to obtain, minimally invasive and a cost‐effective complement that could support and accelerate the diagnostic procedure, especially important for low‐income countries where resources are limited [8]. In Europe, all children with cHL start therapy with two cycles of OEPA chemotherapy regimen. Subsequent treatment is adapted according to disease stage, risk factors and early response assessment, evaluated with positron emission tomography‐computed tomography (PET/CT) at the end of the second OEPA cycle [9].

The early response assessment is a critical timepoint, as it determines whether radiotherapy is administered [9]. However, distinguishing physiological from pathological uptake on PET/CT can be challenging [10] and circulating biomarkers could provide additional objective measures to improve response assessment, thereby reducing the risk of overtreatment and subsequent radiation‐associated toxicity that can severely affect quality of life. Neutropenic fever is a life‐threatening complication of chemotherapy and is common in paediatric HL, especially during the OEPA‐regimen [11, 12]. Developing neutropenic fever can cause treatment delays and dose reductions that may affect outcomes [12]. Identifying patients at high risk of neutropenic fever could improve monitoring and guide preventive treatment measures.

In this study, we applied a systems‐level immunomonitoring approach previously published by us in cell [13] integrating plasma proteins with immune cells, to characterise systemic immune alterations in paediatric cHL. We compared immune composition before and after induction chemotherapy, contrasted findings with other lymphomas and solid tumours, and explored associations with treatment response and neutropenic fever. Our aim was to identify diagnostic and predictive biomarkers to enhance clinical care for children with cHL and provide a comprehensive dataset that can advance development of precision immunotherapy for these children.

## 2 Methods

### 2.1 Study Population

**FIGURE 1:**
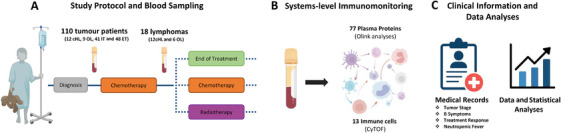
Study outline. (A) Baseline blood samples were collected from 110 children with solid tumours enrolled in the ISAC program (12 cHL, 9 other lymphomas, 41 intracranial and 48 extracranial tumours) prior to treatment initiation, as described in methods. Post‐chemotherapy samples were obtained from 12 children with cHL after two OEPA cycles and six post‐chemotherapy samples from other lymphomas for comparison. (B) Plasma proteins (77) and immune cells (13) were analysed using Olink PEA and CyTOF, respectively. (C) Clinical variables, including tumour stage, B symptoms, early treatment response and neutropenic fever, were extracted from the medical records. Statistical analyses were conducted in R Studio (version 4.5.1). Abbreviations: CHL, classical Hodgkin lymphoma; CT, chemotherapy; ET, extracranial tumour; IT, intracranial tumours; OL, other lymphoma; PEA, proximity extension assay.

This is a sub‐study of the Immune System Against Cancer (ISAC) program, which enrols children < 18 years with solid tumours, treated at Karolinska University Hospital. Written informed consent was obtained from caregivers. Building on our published immune resource [13], we included published and unpublished baseline samples from children with cHL and conducted new comparative analyses against children with other lymphomas, including non‐Hodgkin lymphoma (NHL) and NLPHL, and against those with intra‐ and extracranial tumours. Novel longitudinal post‐induction chemotherapy samples from cHL and other lymphomas were also included (Figure 1).

Baseline samples were collected at diagnosis, before or within 24 h of systemic chemotherapy initiation, or before surgery/radiation. One cHL patient was sampled on Day 2 of induction chemotherapy, and five patients with other lymphomas received intrathecal chemotherapy one day prior to baseline sampling. Treatment details prior to baseline sampling are shown in Figure S1. Baseline clinical data included age, sex, tumour type and absolute count of white blood cells (WBC) and neutrophils (ANC). For cHL and other lymphomas, additional information included tumour stage, B symptoms, early treatment assessment, neutropenic fever, relapse and overall survival.

### 2.2 Staging, Neutropenic Fever and Response Assessment

CHL patients were staged according to Ann Arbor classification. B symptoms were defined as one or more of: fever (> 38.0°C), drenching night sweats or unexplained weight loss > 10% within 6 months [14]. All cHL patients initially received OEPA × 2 and early response assessment was evaluated at the end of the second cycle using PET/CT [10]. Patients with a Deauville score ≥ 4 were classified as inadequate early responders; lower scores were considered adequate. Neutropenic fever was defined as temperature ≥ 38.3°C (or ≥ 38°C >1 h) with ANC < 0.5 × 10^9^/L (or < 1.0 × 10^9^/L if predicted to decrease) during OEPA.

### 2.3 Sample Collection, Plasma Protein Profiling and Mass Cytometry

Peripheral blood was collected into K_2_EDTA vacutainers. Aliquots were stabilised (Cytodelics AB) or mixed with PAXgene (BD Biosciences), incubated, and stored at −80°C. Remaining blood was centrifuged at 2000 *g* for 10 min at 4°C, and plasma was stored at −80°C. Plasma proteins were measured using the Olink AB Target Immune‐Oncology panel. For mass cytometry, whole blood was stabilised, cryopreserved (Cytodelics AB), thawed, fixed/lysed, barcoded, pooled and stained with a metal‐conjugated antibody panel prior to CyTOF XT acquisition. Further details are provided in our previous publication [13].

### 2.4 Outcome and Statistical Analysis

The primary outcome was to characterise systemic immune alterations in paediatric cHL. At baseline, 77 plasma proteins were first compared between cHL and other lymphomas, with significant proteins further assessed against extracranial and intracranial tumours. Associations with age, sex, tumour stage, B symptoms, early treatment response, neutropenic fever and chemotherapy effects were also examined. The WBC, ANC and relative fraction of 13 immune cells and platelets were compared between cHL and the other groups at baseline. The associations between immune cells at baseline and occurrence of neutropenic fever during induction chemotherapy were also evaluated. Group differences were tested using Wilcoxon or Kruskal–Wallis tests, within‐patient changes with paired Wilcoxon and Spearman's rank correlation coefficients for continuous variables. Principal component analysis (PCA) was applied to visualise variance in plasma protein profiles pre‐ and post‐chemotherapy. Analyses were conducted in R (version 4.5.1) with Benjamini–Hochberg adjusted two‐sided *p* values < 0.05 considered significant.

### 2.5 Declaration of Generative AI Technologies

The authors used *GPT‐4/5 (OpenAI)* to improve manuscript clarity and readability. All content was reviewed and edited by the authors, who take full responsibility. No AI‐generated content constitutes novel research findings.

## 3 Results

### 3.1 Patient Characteristics

From the ISAC program [13], we included 13 children with cHL and 11 with other lymphomas, consisting of 4 T‐lymphoblastic, 2 Burkitt, 2 NLPHL, 1 B‐lymphoblastic, 1 anaplastic large cell and 1 peripheral T‐cell lymphoma. In addition, 41 children with intracranial and 48 with extracranial tumours were included. Patient characteristics are summarised in Table 1 and tumour subtype distributions in Table S1A. The cHL cohort had a significantly higher age than the others. Most cHL cases were Stage II (69%), 7/13 had B symptoms, 9/13 had an adequate early response and neutropenic fever occurred in 5/13 cHL patients during the first two OEPA cycles. No cHL patients relapsed and all children with cHL and other lymphomas remain alive as of August 10, 2025, with a median follow‐up of 5.1 years (range 1.9–7.1 years) from time of diagnosis.

**TABLE 1:** Patient demographics and clinical features. (A) Number of patients, age at diagnosis (median and interquartile range) and sex distribution across the whole cohort. Wilcoxon rank‐sum tests were used for statistical comparison of age between classical Hodgkin lymphoma and other tumour groups. (B) Extended clinical information for classical Hodgkin lymphoma and other lymphoma, including tumour stage, presence of B symptoms, early treatment response, neutropenic fever during induction chemotherapy, relapse, and overall survival.

A: Patient Characteristics				
	Classical Hodgkin Lymphoma	Other Lymphoma^a^	Intracranial Tumour	Extracranial Tumour
**N patients**	13	11	41	48
**Age at diagnosis, median (IQR)**	15 (14–17)	10 (6.5–12.5)	9 (5–13)	3 (0.8–8.2)
Age p‐value		0.001**	<0.001***	<0.001***
**Sex**				
Female	8 (61.5%)	4 (36.4%)	15 (36.6%)	25 (52.1%)
Male	5 (38.5%)	7 (63.6%)	26 (63.4%)	23 (47.9%)

B: Clinical Features of Lymphoma Patients		
	Classical Hodgkin Lymphoma	Other Lymphoma
**Tumor Stage**		
Stage I	0 (0%)	1 (9.1%)
Stage II	9 (69.2%)	0 (0%)
Stage III	3 (23.1%)	4 (36.4%)
Stage IV	1 (7.7%)	6 (54.5%)
**B symptoms**		
Yes / No	6 (46.2%) / 7 (53.8%)	3 (27.3%) / 8 (72.7%)
**Early Treatment Assessment**		
Adequate	9 (69.2%)	11 (100%)
Inadequate	4 (30.8%)	0 (0%)
**Neutropenic fever**		
Yes / No	5 (38.5%) / 8 (61.5%)	9 (81.8%) / 2 (18.2%)
**Relapse**	0 (0%)	2 (18.2%)
**Survival (overall)**	13 (100%)	11 (100%)

^a^ Other lymphoma includes following subgroups: T lymphoblastic lymphoma (*n* = 4), nodular lymphocyte predominant Hodgkin lymphoma (*n* = 2), Burkitt lymphoma (*n* = 2), peripheral T cell lymphoma (*n* = 1), anaplastic large cell lymphoma (*n* = 1) and B lymphoblastic lymphoma (*n* = 1).

### 3.2 Baseline Differences in Plasma Proteins and Immune Cell Composition

**FIGURE 2:**
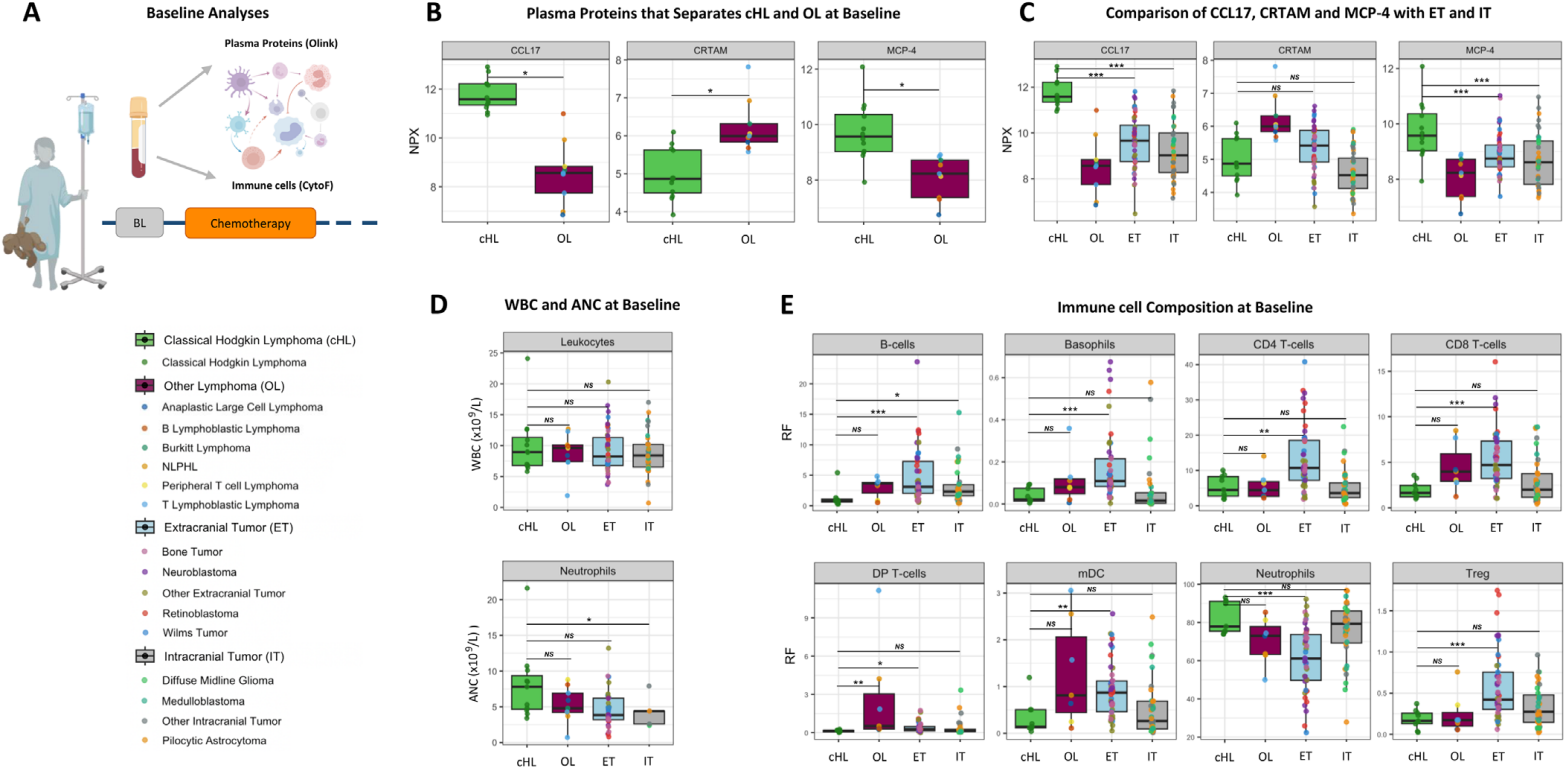
Baseline differences in plasma proteins and immune cell composition. (A) Blood samples were collected from children with cHL, other lymphomas, intra‐, and extracranial tumours at baseline and analysed for 77 plasma proteins and 13 immune cell subsets. One child with cHL and two with other lymphomas had no baseline sample and were excluded from analyses. (B) Plasma proteins that significantly differed between cHL and other lymphomas. (C) Levels of CCL17, MCP‐4 and CRTAM were compared between cHL and intracranial tumours and between cHL and extracranial tumours. (D) Comparison of WBC and ANC at baseline in cHL, other lymphomas, intra‐, and extracranial tumours. (E) Differences in immune cell composition between cHL, other lymphomas, intra‐ and extracranial tumours at baseline. Abbreviations: ANC, absolute neutrophil count; BL, baseline; CHL, classical Hodgkin lymphoma; DP T cells, double positive T cells; ET, extracranial tumour; IT, intracranial tumour; mDC, myeloid dendritic cells; NLPHL, nodular lymphocyte predominant Hodgkin lymphoma; OL, other lymphoma; Treg, regulatory T cells; WBC, white blood cell count. Wilcoxon rank‐sum test was used for all analyses. *p < 0.05; **p < 0.01; ***p < 0.001; NS, not significant.

To identify disease‐specific alterations, we compared 77 plasma proteins between children with cHL and other lymphomas at baseline. After multiple testing correction, three proteins differed significantly (Figure 2B) and 16 additional proteins had an unadjusted *p* < 0.05 (Figure S2). Compared to other lymphomas, cHL had higher levels of C‐C motif chemokine 17 (CCL17/TARC; 9.39‐fold, p = 0.001), monocyte chemoattractant protein‐4 (MCP‐4/CCL13; 3.13‐fold, p = 0.012), and lower levels of CRTAM (2.38‐fold, p = 0.024). We next examined these three proteins in children with intra‐ and extracranial tumours to assess broader disease specificity. CCL17 and MCP‐4 remained significantly higher in cHL compared with both intra‐ and extracranial tumours (*p < *0.0001), supporting their specificity for cHL. In contrast, CRTAM levels in cHL were comparable to both intra‐ and extracranial tumours, indicating that the decrease observed was not disease‐specific for cHL (Figure 2C).

To investigate whether differences in immune cells might underlie the observed protein differences, we first compared WBC and ANC. No significant differences were observed between cHL and other lymphomas, or extracranial tumours; ANC were higher in cHL versus intracranial tumours, although based on a limited number of ANC samples (Figure 2D). Relative fractions of 13 immune cells were then compared (Figure S3) and cHL had lower double positive T‐cells (DP T‐cells) compared to other lymphomas (*p *< 0.05). Compared with intra‐ and extracranial tumours, cHL had lower B‐cell fractions. Compared with extracranial tumours, cHL had lower basophils, CD4+ T‐cells, CD8+ T‐cells, DP T‐cells, myeloid dendritic cells (mDC) and T‐regulatory cells (Treg), and higher neutrophil fractions (Figure 2E).

### 3.3 Association of CCL17 and MCP‐4 Levels With Clinical Variables

**FIGURE 3:**
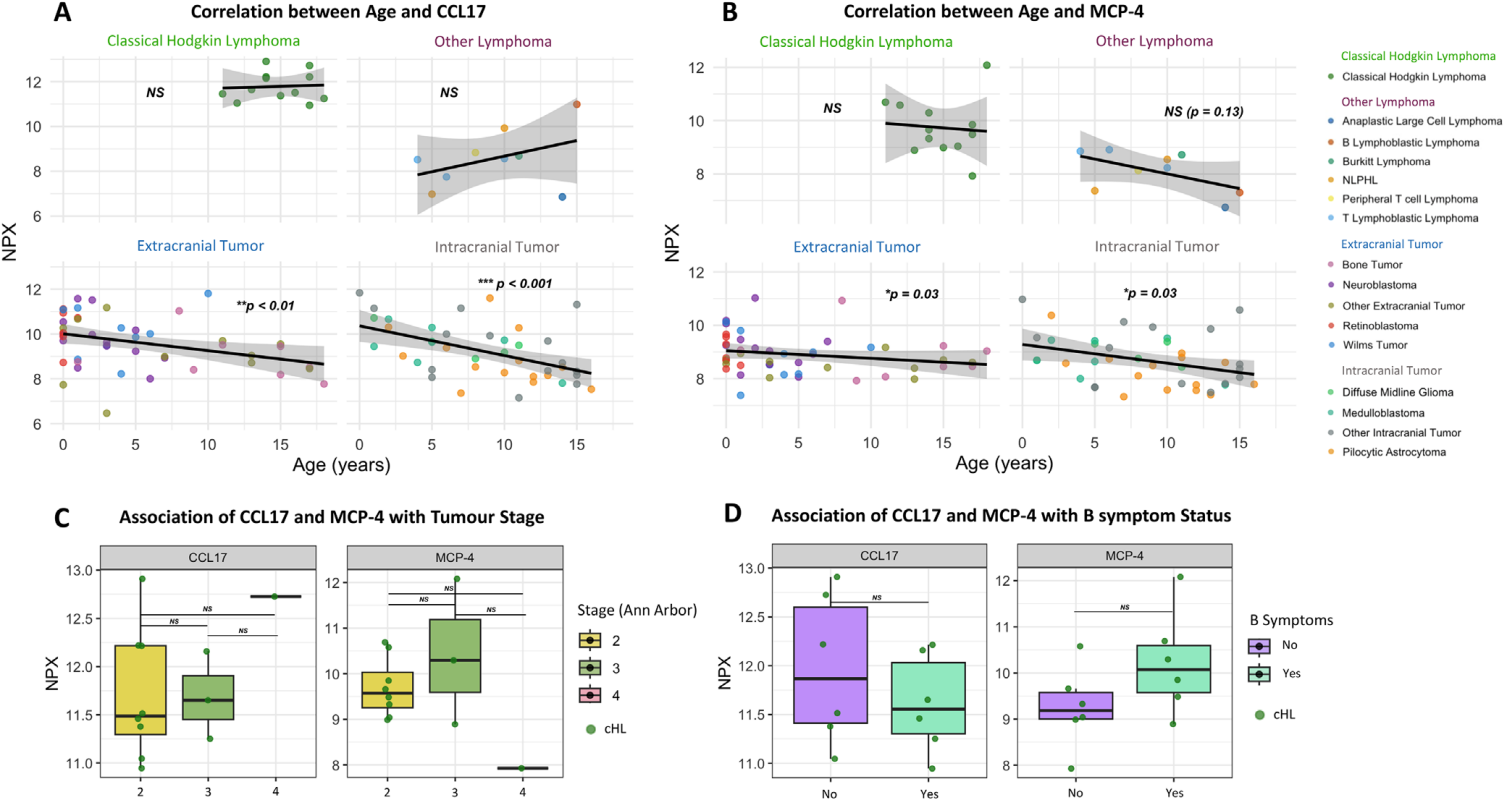
CCL17 and MCP‐4 in relation to age, tumour stage and B symptoms. (A) Spearman correlation between age and CCL17 levels at baseline in cHL, other lymphomas, intra‐ and extracranial tumours. (B) Spearman correlation between age and MCP‐4 levels at baseline in cHL, other lymphomas, intra‐ and extracranial tumours. (C) Association of CCL17 and MCP‐4 levels at baseline with tumour stage (Ann Arbor) in cHL tested using Kruskal–Wallis tests. (D) Association of CCL17 and MCP‐4 levels at baseline with the presence of B symptoms in cHL tested using Wilcoxon rank‐sum tests. Abbreviations: cHL, classical Hodgkin lymphoma; NLPHL, nodular lymphocyte predominant Hodgkin lymphoma. *p < 0.05; **p < 0.01; ***p < 0.001; NS = not significant.

Since CCL17 and MCP‐4 levels were elevated in cHL, we next investigated whether the age differences could explain these results (Table 1). On the contrary, both intra‐ and extracranial cohorts showed negative correlations between age and these protein levels, while no correlation was seen in cHL (Figure 3A,B), indicating that the elevated levels in cHL are not age‐driven. CCL17 and MCP‐4 did not differ by sex in any group (Figure S4), nor by tumour stage in the cHL group. Although the single Stage IV patient had the second‐highest CCL17 and the lowest MCP‐4 levels in the cHL cohort (Figure 3C). No association between CCL17 or MCP‐4 and B symptoms were observed in cHL, but a trend toward higher MCP‐4 levels and B symptoms was observed (Figure 3D).

### 3.4 The Effect of Induction Chemotherapy on Plasma Proteins and Their Association With Early Treatment Response and Neutropenic Fever

**FIGURE 4:**
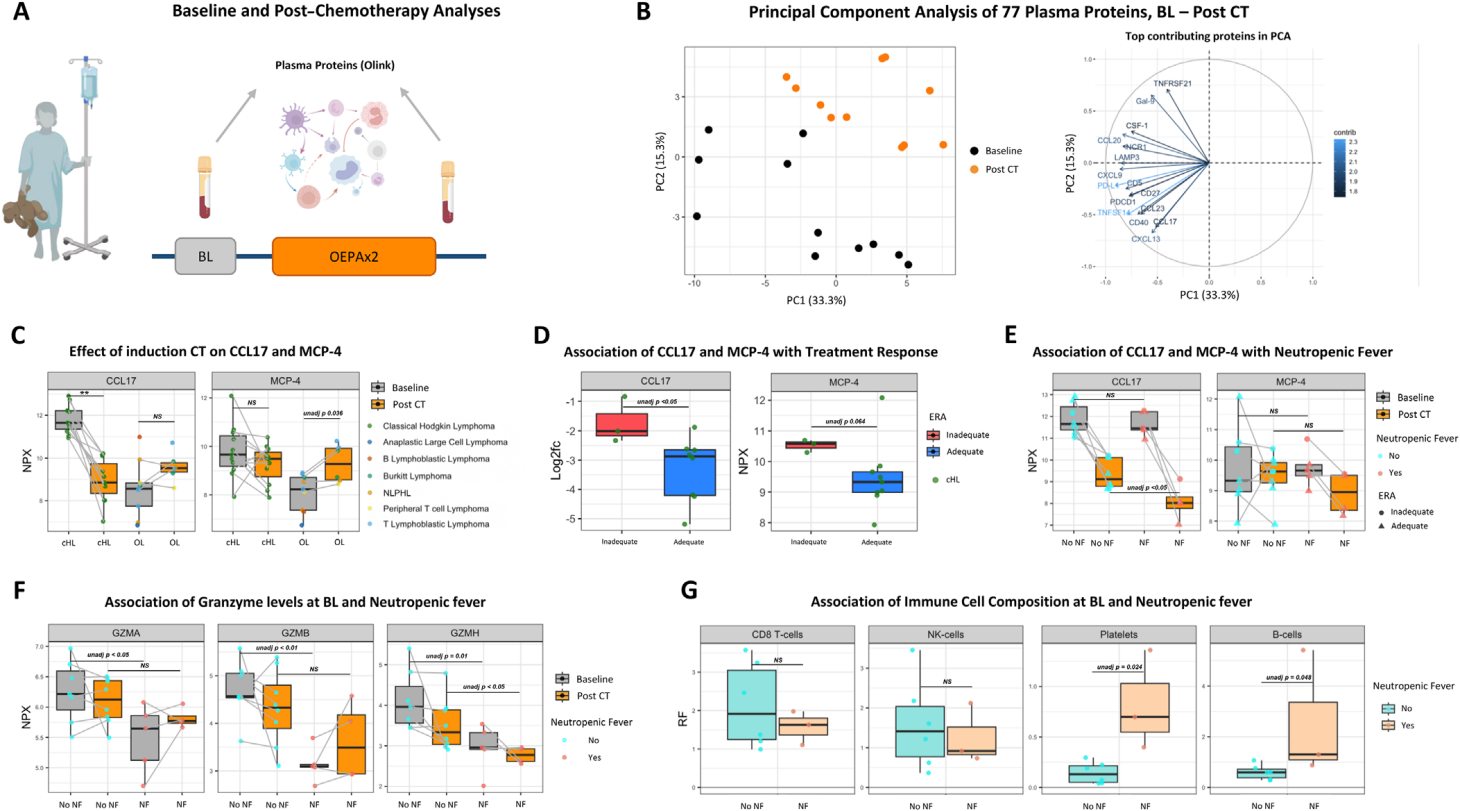
The effect of induction chemotherapy on plasma proteins and their association with early treatment response and neutropenic fever. (A) Blood samples from children with cHL after two cycles of OEPA were analysed for 77 plasma proteins. Post‐chemotherapy samples from other lymphoma patients were also analysed for comparison. (B) A PCA of paired baseline and post‐chemotherapy samples from 11 cHL patients showed clustering of post‐chemotherapy samples, with CCL17 among the top contributors. (C) Changes in CCL17 and MCP‐4 levels in paired cHL samples taken before and after chemotherapy. (D) Association of CCL17 log2fc (baseline–post chemotherapy) and baseline MCP‐4 levels with treatment response at early response assessment in cHL. (E) Association of CCL17 and MCP‐4 at baseline and post‐chemotherapy with the occurrence of neutropenic fever during OEPA × 2 in cHL. (F) Association of Baseline and post‐chemotherapy levels of granzymes GZMA, GZMB and GZMH with the occurrence of neutropenic fever during OEPA × 2 in cHL. (G) Association of immune cell composition at baseline with the occurrence of neutropenic fever during OEPA × 2 in cHL. Abbreviations: BL, baseline; CHL, classical Hodgkin lymphoma; CT, chemotherapy; ERA, early response assessment; Log2fc, log2 fold change; NF, neutropenic fever; NLPHL, nodular lymphocyte predominant Hodgkin lymphoma; PCA, principal component analysis. Statistical tests: Paired Wilcoxon signed‐rank for cHL and Wilcoxon rank‐sum for OL in (C); Wilcoxon rank‐sum (D–G). *p < 0.05; **p < 0.01; ***p < 0.001; NS = non‐significant.

We next analysed plasma proteins, WBC and ANC in children with cHL after two cycles of OEPA (Figure 4A). For comparison, post‐chemotherapy protein levels were also examined in other lymphomas receiving subtype‐specific chemotherapy (Table S1B). A PCA of paired cHL samples showed clustering of post‐chemotherapy samples, indicating a shared plasma protein profile, with CCL17 being a top contributor (Figure 4B).

In cHL, 18 proteins decreased and seven increased following chemotherapy (Figure S5A,B). Compared with other lymphomas, most trends were similar, but CCL17 and MCP‐4 decreased in cHL while showing an apparent increase in other lymphomas (Figure 4C). In cHL, CCL17 significantly decreased (*p *< 0.01), whereas MCP‐4 showed no overall change, though 9/11 patients declined in MCP‐4 (median log2FC of −0.67, IQR −1.29 to −0.01). WBC and ANC decreased after OEPA, confirming myelotoxicity (Figure S5C).

CCL17 decreased in all 11 cHL patients, but with a greater reduction in adequate responders (unadjusted *p* < 0.05) and higher baseline MCP‐4 showed association with inadequate response (unadjusted *p* = 0.064) (Figure 4D). The inadequate responders also had a greater increase in CD83 and TNFRSF21 (unadjusted *p* < 0.05) (Figure S5D). Among cHL patients, five developed neutropenic fever during OEPA × 2 and eight did not. Post‐chemotherapy CCL17 were lower in neutropenic fever patients (unadjusted *p* < 0.05), whereas MCP‐4 showed no association (Figure 4E). Baseline reductions in eight proteins, including GZMA, GZMB and GZMH, were observed in neutropenic fever patients (Figure 4F). Platelets and B‐cell fractions were higher at baseline in the neutropenic fever group (unadjusted *p* < 0.05), while CD8+ T‐cells and NK‐cells showed no differences (Figure 4G).

Post‐chemotherapy, 16 proteins differed between the groups, with 15 reduced in neutropenic fever patients, including immune checkpoints PD‐L2 and PDCD1 (Figure S6A). Despite these differences, WBC and ANC were similar pre‐ and post‐chemotherapy (Figure S6B). Four neutropenic fever patients had adequate early response and one inadequate, compared with three inadequate responses in the non‐neutropenic fever group (Fisher's exact test, *p* = 1) (Table S1B). Notably, the single neutropenic fever patient with inadequate response had the highest CCL17 level in the group post‐chemotherapy (Figure 4E). None of the neutropenic fever‐associated protein differences remained significant after correction for multiple testing.

## 4 Discussion

Using systems‐level immune monitoring, we identified CCL17 and MCP‐4 as potential diagnostic biomarkers distinguishing cHL from other paediatric tumours. In addition, cHL patients differed in immune cell composition, mainly to extracranial tumours, with lower basophil, CD4+, CD8+ and DP T‐cells, mDC and Treg fractions alongside higher neutrophil fractions. CCL17 and MCP‐4 levels negatively correlated with age in intra‐ and extracranial tumours but not in cHL, indicating disease‐specific regulation. Post‐chemotherapy, protein profiles were similar across the cHL cohort and pre‐treatment differences in CCL17 and MCP‐4 between cHL and other lymphomas were eliminated. We also found a suggestive association of CCL17 and MCP‐4 with inadequate early response and reduced granzyme levels in patients who developed neutropenic fever, providing new insights into treatment‐related risks.

CCL17 is a chemokine produced by several cell types, including HRS cells, and it has been speculated that CCL17 drives an influx of T‐helper type 2 cells that surround HRS cells and impede effective immune responses [15, 16]. Elevated CCL17 levels have previously been reported in both adult and paediatric cHL [15, 17, 18, 19, 20]. Here, we extend these observations by showing that CCL17 is also specifically elevated compared with other paediatric lymphomas and solid tumours, highlighting its clinical relevance in the paediatric oncology setting. A negative correlation between age and CCL17 levels has been described [19] as well as a decrease in CCL17 following induction chemotherapy in HL patients [21, 22]. Surprisingly, other lymphoma patients showed higher median CCL17 levels after chemotherapy, indicating that the decline observed in cHL is not merely an immunosuppressive effect of chemotherapy, but rather reflects suppression of disease‐related overproduction.

In adult HL, high baseline CCL17 levels have been associated with poor treatment response [18]. While we did not observe this association, we found that children with adequate early response displayed a larger decline in CCL17 than inadequate responders, underscoring the importance of longitudinal monitoring. Furthermore, we observed lower CCL17 levels after chemotherapy in children who developed neutropenic fever. Although 4/5 neutropenic fever patients showed an adequate early response compared with three inadequate responders in the non‐neutropenic fever group, this difference was not significant, and it remains unclear whether the lower CCL17 levels in neutropenic fever patients reflect a stronger treatment effect or greater myelotoxicity. These observations do however, indicate that neutropenic fever could influence CCL17 levels independently of treatment efficacy, representing a potential confounding factor when considering CCL17 as a biomarker of treatment response.

MCP‐4 promotes immune cell migration and proinflammatory cytokine secretion [23, 24] and is expressed by various cell types, including HRS cells [25]. Here, we demonstrate that plasma MCP‐4 levels are elevated in paediatric cHL at diagnosis. Previous reports have been inconsistent. Agrusa et al. found no diagnostic difference between paediatric HL and healthy controls but noted that higher pre‐therapy MCP‐4 levels predicted slower early response, aligning with our observation of elevated baseline MCP‐4 levels and inadequate early response [20]. Gholila et al. instead observed increased MCP‐4 levels in cHL tissue, but not plasma, in a mixed paediatric and adult cohort [26]. Thus, our findings provide novel evidence on elevated levels of plasma MCP‐4 in paediatric cHL compared to other childhood lymphomas and solid tumours. Following chemotherapy, MCP‐4 levels decreased in most cHL patients, contrasting with chemotherapy changes in OL.

Importantly, while overall survival is excellent in paediatric cHL and no relapses were observed in our cohort, early response remains a key clinical endpoint, as it directly determines whether radiotherapy should be administered [9]. Biomarkers such as CCL17 and MCP‐4 could complement imaging, improve precision of early response assessment and potentially reduce the risk of under‐ or overtreatment. Although exploratory, these findings highlight the potential of CCL17 and MCP‐4 to refine diagnosis and monitor treatment response in paediatric cHL.

Neutropenic fever is a common and serious complication of paediatric chemotherapy [12]. In our cohort, non‐neutropenic fever patients had higher baseline levels of GZMA, B and H, which are key mediators of cytotoxic immune responses [27], suggesting a more activated cytotoxic state that may reduce susceptibility to infections during chemotherapy. The patients who developed neutropenic fever showed broadly lower protein levels after chemotherapy despite comparable WBC and ANC, indicating an immune suppression not captured by standard clinical measures. These findings suggest that plasma protein profiling could identify children at higher neutropenic fever risk, although validation in larger, independent cohorts are required.

Chemotherapy is known for its myelo‐ and lymphotoxic effects, yet accumulating evidence also highlights its immune‐stimulatory properties, showing that chemotherapy can trigger a tumour‐specific immune responses and enhance immunotherapy [28, 29, 30]. The efficacy of such combinations likely depends on dose, timing and sequence, underscoring the need to evaluate each regimen individually [30]. Here, we characterised the impact of induction chemotherapy on 77 plasma proteins in paediatric cHL and found significant changes in 25 proteins with seven increasing. This may reflect immune stimulation or relief from immunosuppression, suggesting that OEPA‐regimen could create an immunologic milieu favourable for combination with immunotherapy.

Although our cohort is relatively large compared with other similar paediatric studies, it is still underpowered for detailed analyses within the cHL subgroup. The limited sample size hampers generalizability of our findings and precludes formal validation in an independent cohort. Another limitation is that five other lymphomas received intrathecal chemotherapy the day before baseline, with unknown effects on plasma protein. However, no consistent trends in MCP‐4 or CCL17 were observed when comparing these patients with untreated other lymphomas. In addition, one cHL and two other lymphomas received corticosteroids prior to baseline, which may influence immune composition. Corticosteroid pre‐treatment is also common in children with intracranial tumours and could similarly affect immune profiles. Despite these limitations, including paediatric patients with other lymphomas and solid tumours provides a novel, clinically relevant, paediatric‐specific context. While not a substitute for an independent validation cohort, these comparisons offer insights into systemic immune alterations that can guide future research.

## 5 Conclusion

In conclusion, we show that CCL17 and MCP‐4 are specifically elevated in paediatric cHL compared with other childhood lymphomas and solid tumours, suggesting their potential as diagnostic and response biomarkers. In addition, lower granzyme levels were associated with neutropenic fever, pointing to the importance of immune profiling in clinical practice for risk stratification. Our characterisation of the immune composition at time of diagnosis and the modulatory effects of induction chemotherapy could also aid in optimising future combinations with precision immunotherapy. Together, these findings highlight clinically relevant biomarkers in a paediatric oncology context that can enhance diagnostic precision, guide response‐adapted therapy and effectively allocate supportive care, thereby improving outcomes for children with cHL.

## Author Contributions

G.H designed this sub‐study, and P.B., L.L. and P.K. designed the larger ISAC‐study and established the study protocol. T.L. and P.B. established experimental protocols and T.L. conducted experimental analyses. Q.C performed raw data analyses. N.H contributed with clinical input. G.H. collected clinical data, performed data analyses and wrote the paper, with input from all co‐authors.

## Funding

This study, and the ISAC‐program, was possible thanks to funding from the Swedish Childhood Cancer Fund (PR2022‐0114, PR2019‐0070 and PR2017‐0103 to P.B., PR2020‐0133, TJ2016‐0058 and TJ2019‐0111 to P.K.; and KF2023‐0004 to L.L.), Swedish Research Council (2019‐01495, 2020‐06190, 2020‐02889, 2021‐06529, 2021‐05450 and 2022‐01567 to P.B.), the Swedish Cancer Society (201175PjF, CAN 2015/587 and CAN 2018/764 to P.B.), Karolinska Institutet (2018‐02229), Göran Gustafsson Foundation (GG2020‐0040), Knut & Alice Wallenberg Foundation (KAW2023‐0344 and 2019.0191), and the Swedish Society for Medical Research (CG‐22‐0148‐H‐02). The funding sources of this study had no role in study design, data collection, analysis, interpretation, manuscript writing, or the decision to submit. The first draft was written by Dr Hedberg, and no honorarium or payment was received.

## Ethics Statement

Written informed consent was obtained from the caregivers and the study was approved by the Regional Ethics Committee in Stockholm, Sweden (EPM: 2017/2055‐31, 2020‐07090, 2021‐ 05571‐02 and 2024‐00817‐02). All conducted in accordance with the Declaration of Helsinki.

## Conflicts of Interest

Petter Brodin and Tadepally Lakshmikanth are co‐founders of Cytodelics AB. Petter Brodin is a scientific advisor to Pixelgen Technologies AB (Stockholm, Sweden), Sention Health AB (Stockholm, Sweden), Helaina Inc. (New York, US), Scailyte AG (Basel, CZ), and Oxford Immune Algorithmics (Reading, UK). The other authors declare no conflicts of interest.

## Supporting information



Supporting Information: jha270228‐sup‐0001‐SuppMat.pdf

## Data Availability

Plasma protein data are available upon request following relevant material transfer procedures. Preprocessed immunological data are shared at GitHub: https://github.com/Brodinlab/ISAC1. Mass cytometry raw data (FCS files) are deposited in FlowRepository.org: http://flowrepository.org/ with ID: FR‐FCM‐Z8C8. Requests for resources and further information should be directed to senior author Linda Ljungblad.
