# Supplementary material for: Systemic Immune Alterations in Paediatric Classical Hodgkin Lymphoma With CCL17 and MCP‐4 as Diagnostic and Predictive Biomarkers
Source: EJHaem. 2026 Jan 28;7(1):e70228. doi: 10.1002/jha2.70228 (PMC12849930; doi:10.1002/jha2.70228)
Supplement: Supplementary file 1 — Supporting Information: jha270228‐sup‐0001‐SuppMat.pdf [file JHA2-7-e70228-s001.pdf]

Supplementary Figures and Tables

Systemic immune alterations in pediatric Hodgkin lymphoma with CCL17 and MCP-4 as diagnostic and predictive biomarkers

Gustav Hedberg, Qi Chen, Tadepally Lakshmikanth, Nikolas Herold, Per Kogner, Petter Brodin, Linda Ljungblad

Figures

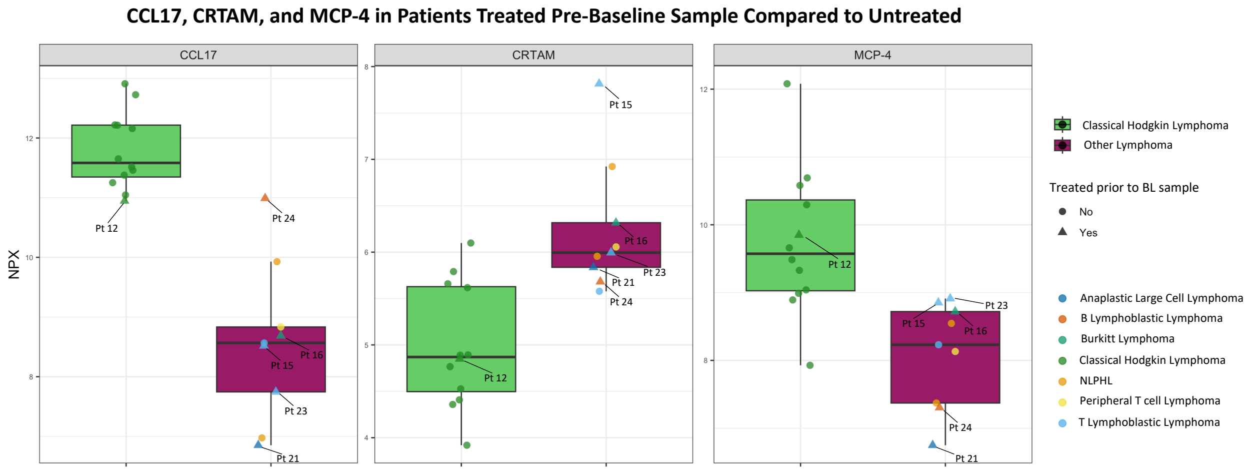

Figure S1. Treatment details prior baseline sampling and their CCL17, CRTAM and MCP-4 levels in relation to untreated patients.

Pt 12 received day 1 of the OEPA-regimen prior to baseline sample.

Pt 15 received Intra thecal methotrexate the day before baseline sample.

Pt 16 received Intra thecal methotrexate, cytarabine and prednisolon the day before baseline sample.

Pt 21 received Intra thecal methotrexate, cytarabine and prednisolon the day before baseline sample and methylprednisolon during 2 days prior to baseline samples (4 doses i.v.)

Pt 23 received Intra thecal methotrexate the day before baseline sample.

Pt 24 received Intra thecal methotrexate and 1 dose of betamethasone the day before baseline sample.

Abbreviations: Pt, patient; BL, baseline; NLPHL, Nodular lymphocyte predominant Hodgkin lymphoma.

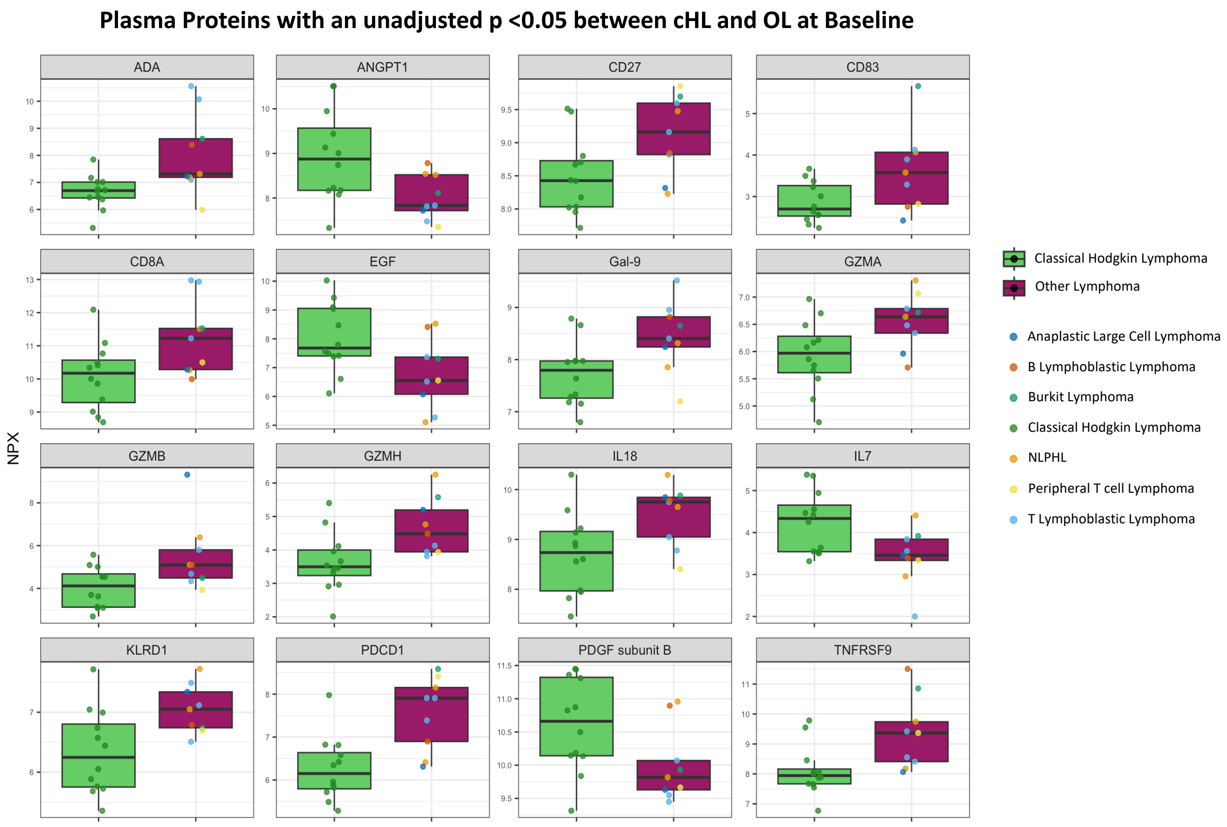

Figure S2. Boxplot of plasma proteins that had an unadjusted p<0.05 between cHL and other lymphomas at baseline but that didn't reach statistical significance after correction for multiple testing

Abbreviations: CHL, classical Hodgkin lymphoma; NLPHL, nodular lymphocyte predominant Hodgkin lymphoma.

Wilcoxon rank-sum test was used for all analyses above, adj p-value method Benjamin-Hochberg

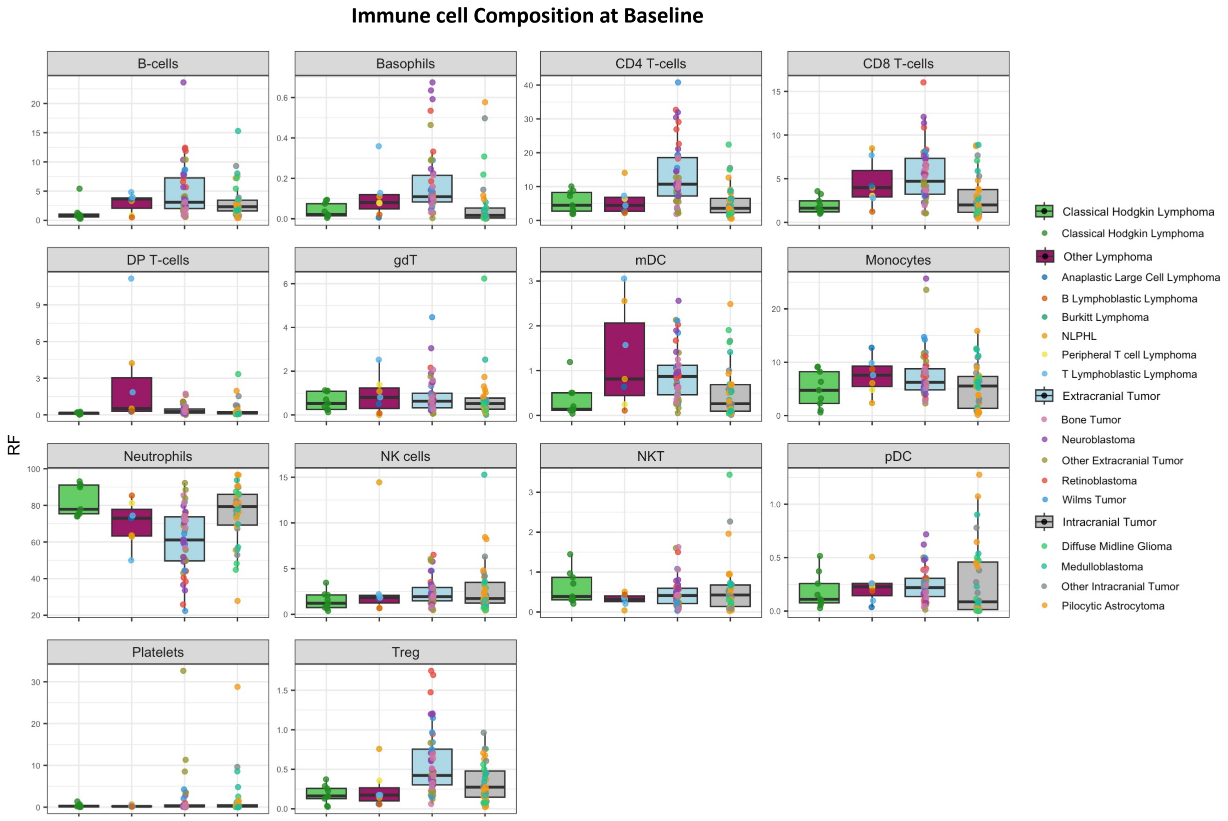

Figure S3, Relative fraction of all measured immune cells and platelets at baseline in Classical Hodgkin Lymphoma, Other Lymphoma, Extracranial Tumors and Intracranial tumors

Abbreviations: DP T-cells, Double positive T-cells; gdT, Gamma delta T-cells; mDC, myeloid Dendritic cells; NK cells, Natural killer cells; NKT, Natural killer T-cells; pDC, Plasmacytoid Dendritic cells; Treg, T regulatory cells; NLPHL, nodular lymphocyte predominant Hodgkin lymphoma.

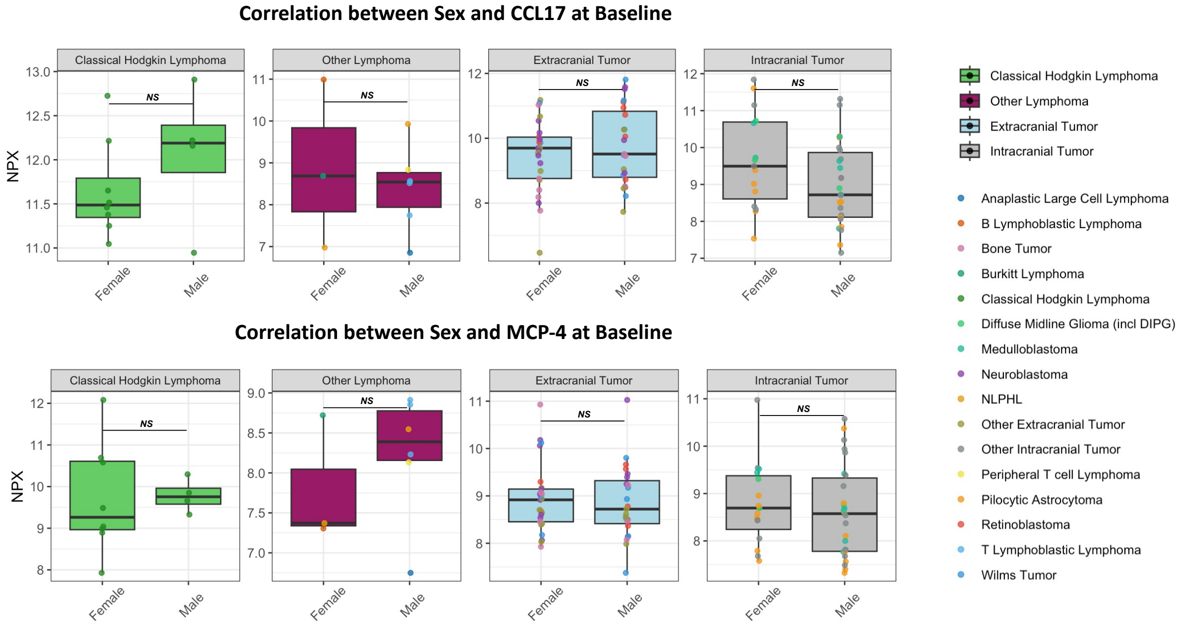

Figure S4, Correlation of sex with baseline CCL17 and MCP-4 levels in the different tumor groups.

Abbreviations: NS, not significant; NLPHL, Nodular lymphocyte predominant Hodgkin lymphoma, DIPG, Diffuse intrinsic pontine glioma.

Wilcoxon rank-sum test was used for all analyses above, adj p-value method Benjamin-Hochberg.

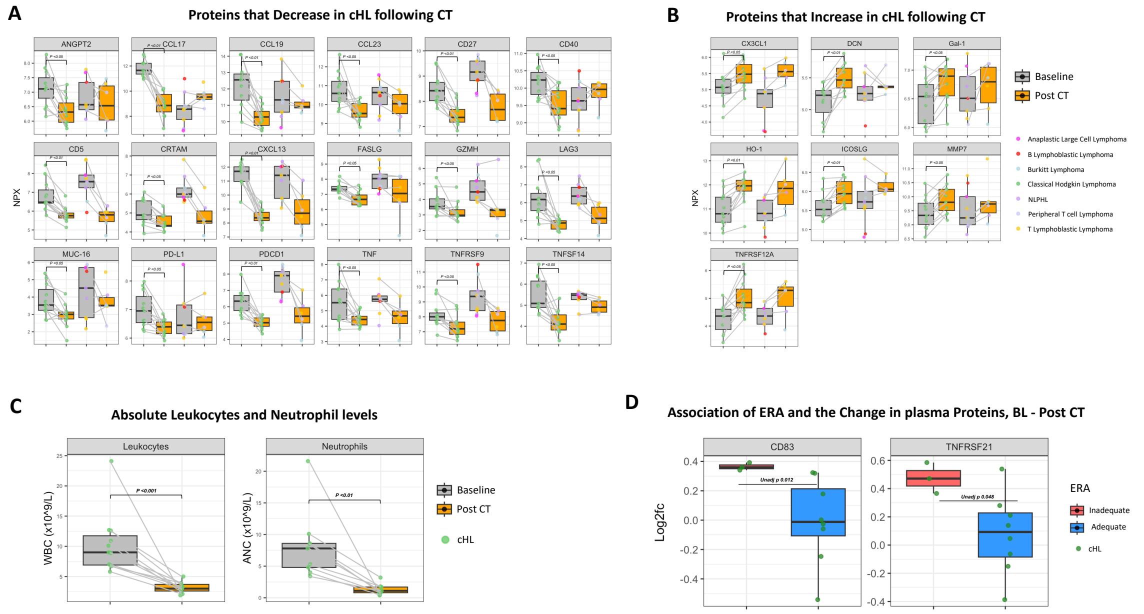

Figure S5, Effect of induction CT (OEPAx2) on plasma protein levels, WBC and ANC in cHL and the association of ERA and the change in CD83 and TNFRSF21, BL – Post chemotherapy.

A) Boxplot of proteins that decrease ($p<0.05$) in 11 children with cHL with paired samples at BL-Post CT. The change in other lymphomas was added for comparisons, none of the proteins significantly differed in the other lymphoma cohort after adjusting for multiple testing.

B) Boxplot of proteins that increase ($p<0.05$) in 11 children with cHL with paired samples at BL-Post CT. The change in other lymphomas was added for comparisons, none of the proteins significantly differed in the other lymphoma cohort after adjusting for multiple testing.

C) Boxplot of WBC and ANC in 11 children with cHL with paired samples at BL and Post-chemotherapy

D) The log2fc in CD83 and TNFRSF21 in relation to early response assessment, between baseline – Post chemotherapy (OEPAx2) in cHL.

Abbreviations: BL, baseline; WBC, White blood cell count; ANC, Absolute neutrophil count; CT, Chemotherapy; cHL, classical Hodgkin lymphoma; PCA, CT, Chemotherapy; NLPHL, nodular lymphocyte predominant Hodgkin lymphoma; ERA, early response assessment; Log2fc, (Log2 fold change).

Statistical tests: paired Wilcoxon signed-rank for cHL in A-C and Wilcoxon rank-sum for OL in A-B and for D, *adj p-value method Benjamin-Hochberg for all analyses.*

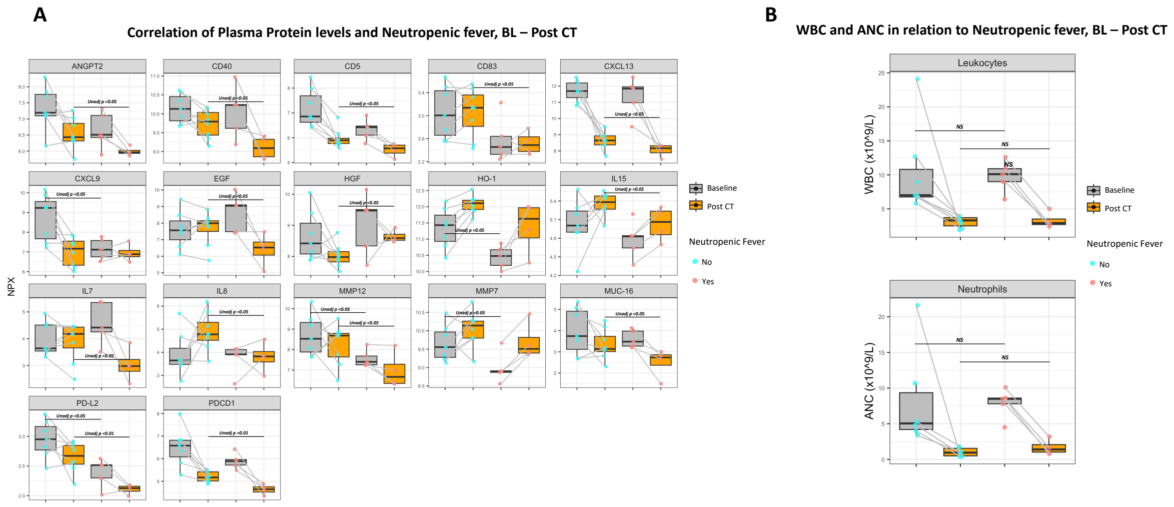

Figure S6, Correlation of plasma protein levels, WBC and ANC and neutropenic fever at BL and Post-chemotherapy in cHL.

A) Correlation of plasma proteins (unadjusted p <0.05) and neutropenic fever at BL and post-chemotherapy in cHL (The proteins published in the manuscript are excluded from this graph)

B) Correlation of WBC and ANC levels in relation to neutropenic fever in cHL, BL - Post chemotherapy.

Abbreviations: BL, Baseline; CT, Chemotherapy; CHL, classical Hodgkin lymphoma; WBS, White blood cell count; ANC, Absolute neutrophil count; NS, not significant.

Wilcoxon rank-sum test was used for all analyses above, adj p-value method Benjamin-Hochberg.

Tables

A

Classical Hodgkin lymphoma (n=13)	Other lymphoma (n=11)	Extracranial Tumor (n=48)	Intracranial Tumor (n=41)
Classical Hodgkin lymphoma (n =13)	Anaplastic Large Cell Lymphoma (n=1)	Bone Tumor (n=8)	Anaplastic Ependymoma (n=1)
	B Lymphoblastic Lymphoma (n=1)	HCC (n=3)	Atypical Teratoid/Rhabdoid Tumor (n=1)
	Burkitt Lymphoma (n=2)	Neuroblastoma HR (n=7)	Diffuse Astrocytoma (1)
	NLPHL (n=2)	Neuroblastoma LR (n=6)	Diffuse Midline Glioma (incl DIPG) (n=5)
	Peripheral T cell Lymphoma (n=1)	Other (n=4)	Ganglioglioma (n=2)
	T Lymphoblastic Lymphoma (n=4)	Retinoblastoma (n=7)	Glioblastoma Multiforme (n=2)
		Rhabdomyosarcoma (n=4)	Medulloblastoma (n=7)
		Wilms Tumor (n=9)	Meningioma (n=4)
			Optical Pathway Glioma (n=1)
			Other Embryonal Tumors (n=2)
			Pilocytic Astrocytoma (n=15)

B

Patient	Tumor (Stage)	Age	Sex	Chemotherapy Agents and Treatment Protocol	Days of Treatment	Days from last dose of CT to Post-CT Sample	Treatment Response (ERA)	B symptoms	Neutropenic fever	Relapse	Survival
1	cHL (IIA)	14	Male	Etoposide, Vincristine, Doxorubicin (EURO-NET-PHL-C1)	42	20	Adequate	No	Yes	No	Yes
2	cHL (IIB)	17	Female	Etoposide, Vincristine, Doxorubicin (EURO-NET-PHL-interim)	45	16	Adequate	Yes	Yes	No	Yes
3	cHL (IVA)	17	Female	Etoposide, Vincristine, Doxorubicin (EURO-NET-PHL-C1)	42	14	Adequate	No	No	No	Yes
4	cHL (IIIB)	14	Male	Etoposide, Vincristine, Doxorubicin (EURO-NET-PHL-interim)	48	14	Inadequate	Yes	No	No	Yes
5	cHL (IIA)	14	Male	Etoposide, Vincristine, Doxorubicin (EURO-NET-PHL-C2)	44	18	Adequate	No	No	No	Yes
6	cHL (IIA)	17	Male	Etoposide, Vincristine, Doxorubicin (EURO-NET-PHL-C2)	42	14	Inadequate	No	No	No	Yes
7	cHL (IIIB)	18	Female	Etoposide, Vincristine, Doxorubicin (EURO-NET-PHL-C2)	52	15	Adequate	Yes	No	No	Yes
8	cHL (IIA)	16	Female	Etoposide, Vincristine, Doxorubicin (EURO-NET-PHL-C2)	42	17	Adequate	No	No	No	Yes
9	cHL (IIA)	12	Female	Etoposide, Vincristine, Doxorubicin (EURO-NET-PHL-C2)	42	16	Inadequate	No	No	No	Yes
10	cHL (IIIB)	13	Female	Etoposide, Vincristine, Doxorubicin (EURO-NET-PHL-C2)	46	16	Adequate	Yes	No	No	Yes
11	cHL (IIB)	11	Female	Etoposide, Vincristine, Doxorubicin (EURO-NET-PHL-C2)	40	11	Inadequate	Yes	Yes	No	Yes
12	cHL (IIB)	17	Male	Etoposide, Vincristine, Doxorubicin (EURO-NET-PHL-C2)	43	14	Adequate	Yes	Yes	No	Yes
13	cHL (IIA)	15	Female	(EURO-NET-PHL-C2)	NA	NA	Adequate	No	Yes	No	Yes
14	NLP-HL (I)	10	M	Cyclophosphamide, Vinblastin (EuroNet-PHL-LP1)	45	23	Adequate	No	No	No	Yes
15	T-LBL (IV)	4	M	MTX IT, Vincristine, Daunorubicin PEG-asparaginas, Cyclophosphamide, 6-merkaptopurin, Cytarabin (EURO-LB-02)	42	3	Adequate	No	Yes	No	Yes
16	Burkitt lymphoma (III)	11	F	MTX + Cytarabin IT, Cyclophosphamide, Rituximab, Vincristine, MTX, Ifosfamide, Cytarabine, Etoposide, Doxorubicin (B-NHL 2013)	84	1	Adequate	No	Yes	No	Yes
17	Peripheral T cell lymphoma (IV)	8	M	MTX + Cytarabin IT, Cyclophosphamide, Vinblastine, Ifosfamid, MTX, Etoposide, Cytarabin, Doxorubicin (BFM-protocol)	52	21	Adequate	No	Yes	No	Yes
18	T-LBL (III)	14	F	Cyclophosphamide, Vincristine, Daunorubicin, MTX IT, Cytarabine (Euro LB-02)	101	21	Adequate	No	No	No	Yes
19	NLPHL (IV)	5	F	HL-C1 + Rituximab	NA	NA	Adequate	No	Yes	No	Yes
20	Burkitt lymphoma (III)	7	M	Vincristine, MTX, Ifosfamid, Cytarabin, Etoposid, Cyklophosfamide, Rituximab, MTX + Cytarabin IT (B-NHL 2013)	53	4	Adequate	No	Yes	No	Yes
21	ALCL (IV)	14	M	ALCL 99	NA	NA	Adequate	Yes	Yes	Yes	Yes
22	T-LBL (IV)	10	M	LBL-2018	NA	NA	Adequate	No	Yes	No	Yes
23	T-LBL (IV)	6	M	LBL-2018	NA	NA	Adequate	Yes	Yes	No	Yes
24	B-LBL (III)	15	F	DA-EPOCH-R	NA	NA	Adequate	Yes	Yes	Yes	Yes

Table S1. Tumor Subtypes, patient characteristics, disease and treatment details.

A) Tumor subtypes distribution in each cHL, other lymphomas, intra- and extracranial tumours.

B) Detailed information on each patient's characteristics, disease subtype and treatment in cHL and other lymphomas. The column "Chemotherapy Agents and Treatment Protocol" lists the specific drugs and protocol each patient received between baseline and post chemotherapy samples. The column "Days of Treatment" indicated the number of days each patient received chemotherapy during this period.

Abbreviations: HCC = Hepatocellular carcinoma, HR = High Risk, LR = Low Risk, DIPG = Diffuse Intrinsic Pontine Glioma; ALCL, Anaplastic large cell lymphoma; LBL, Lymphoblastic lymphoma; NLPHL, Nodular lymphocyte predominant Hodgkin lymphoma; cHL, classical Hodgkin lymphoma; ERA, early response assessment; NA, not applicable due to lack of post CT sample.
